# Alterations in neuroblastoma ganglioside synthesis by induction of GD1b synthase by retinoic acid

**DOI:** 10.1038/sj.bjc.6601914

**Published:** 2004-06-08

**Authors:** S Hettmer, R McCarter, S Ladisch, K Kaucic

**Affiliations:** 1 Glycobiology Program, Center for Cancer and Immunology Research, Children's National Medical Center, 111 Michigan Avenue, NW, Washington DC 20010, USA; 2 Biostatistics and Informatics Unit, Center for Health Services and Community Research, Children's National Medical Center, 111 Michigan Avenue, NW, Washington DC 20010, USA; 3 Department of Pediatrics, George Washington University School of Medicine and Health Sciences, Washington DC 20010, USA

**Keywords:** neuroblastoma, gangliosides, retinoic acid, GD1b synthase

## Abstract

Recent findings link increased expression of the structurally complex ‘b’ pathway gangliosides GD1b, GT1b, GQ1b (CbG) to a favourable clinical and biological behaviour in human neuroblastoma (NB). Seeking a model to probe these observations, we evaluated four human NB cell lines. Very low CbG content (4–10%) in three of the four cell lines (LAN-5, LAN-1, SMS-KCNR) reflected the ganglioside pattern observed in the most aggressive NB tumours. Pharmacological alterations of complex ganglioside synthesis *in vitro* by a 5–7 day exposure to 5–10 *μ*M retinoic acid, which is employed in maintenance therapy of disseminated NB, included markedly increased (i) relative expression of CbG (6.6±2.0-fold increase, *P*=0.037), (ii) relative expression of the analogous ‘a’ pathway gangliosides, termed CaG (6.4±1.4-fold increase in GM1a and GD1a; *P*=0.010), and (iii) total cellular ganglioside content (2.0–6.3-fold), which in turn amplified the accumulation of structurally complex gangliosides. Substantial increases (2.7–2.9-fold) in the activity of GD1b/GM1a synthase (*β*-1,3-galactosyltransferase), which initiates the synthesis of CbG and CaG, accompanied the all-*trans* retinoic acid (ATRA)-induced ganglioside changes. Thus, increased CbG synthesis in NB cell lines is attributable to a specific effect of ATRA, namely induction of GD1b/GM1a synthase activity. Since the shift towards higher expression of CbG and CaG during retinoic acid-induced cellular differentiation reflects a ganglioside pattern found in clinically less-aggressive tumours, our studies suggest that complex gangliosides may play a role in the biological and clinical behaviour of NB.

Increasing evidence has implicated gangliosides, a specific class of cell surface glycosphingolipids, in the biological and clinical behaviour of many types of tumours including human neuroblastoma (NB). Gangliosides are overexpressed and actively shed by tumour cells (Wu *et al*, 1986; Valentino *et al*, 1990; Li and Ladisch, 1991) and have a number of biological properties that could conceivably alter tumour–host interactions to influence the survival of the malignant cells that carry these molecules (Hakomori, 1996). Our previous studies have linked specific ganglioside changes in human NB tumours to differences in the clinical and biological behaviour of this tumour (Kaucic *et al*, 2001; Hettmer *et al*, 2003), and raise the possibility that pharmacologically induced modulation of NB ganglioside content could have important consequences for outcome.

Gangliosides consist of a sialic acid-containing carbohydrate portion and a lipid portion (ceramide) embedded in the outer leaflet of the cell membrane. They are synthesised via two prominent pathways, designated ‘a’ (GM2, GM1a, GD1a) and ‘b’ (GD3, GD2, GD1b GT1b, GQ1b), from a common precursor (GM3) derived from lactosylceramide (Figure 1

**Figure 1:**
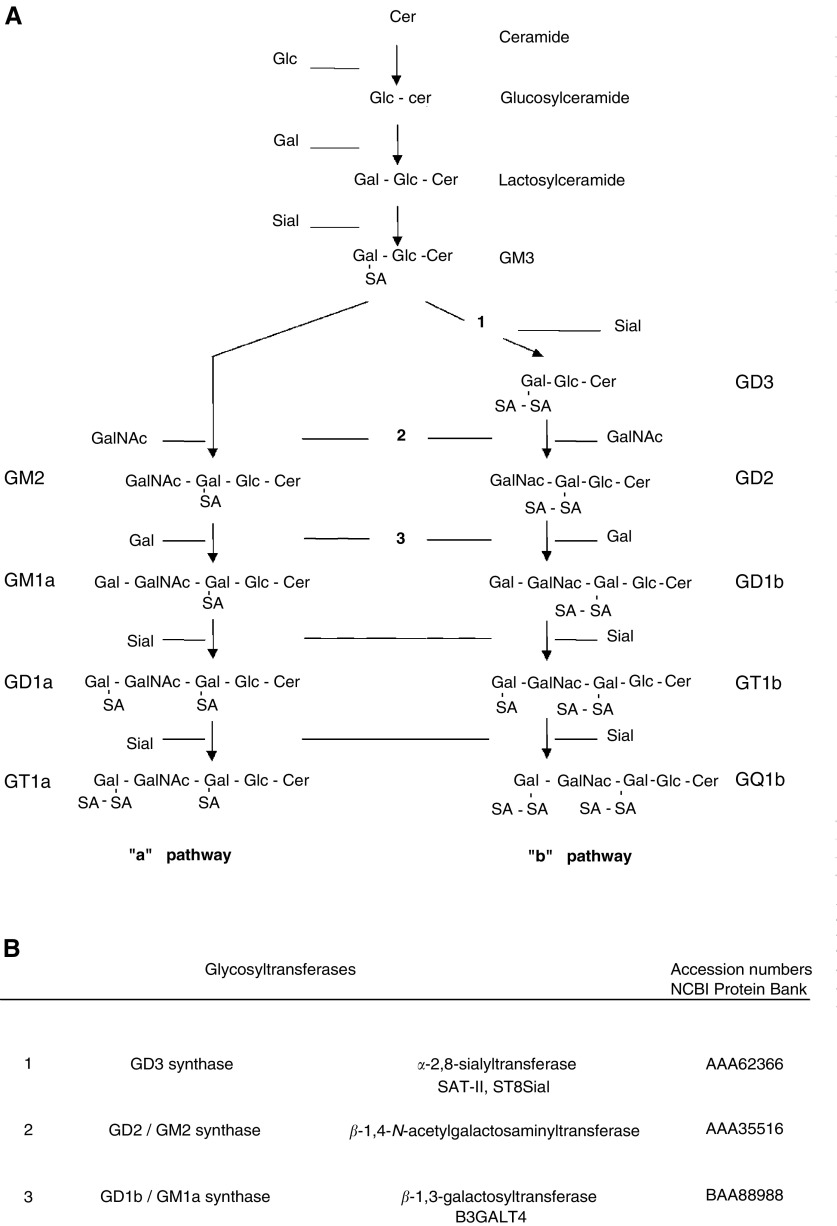
Schematic representation of the major pathways of ganglioside biosynthesis. (**A**) The monosialoganglioside GM3, derived from lactosylceramide, is the common precursor for both ‘a’ and ‘b’ pathway gangliosides. Each ganglioside species consists of a ceramide backbone (CER), and a carbohydrate chain (glc=glucose, gal=galactose, GalNAc=*N*-acetylgalactosamine) containing one or more sialic acid (SA) residues. ‘a’ and ‘b’ pathway gangliosides downstream of GD1b/GM1a synthase were designated complex ‘a’ (CaG) and complex ‘b’ (CbG) gangliosides, respectively. (**B**) Parallel steps in both pathways are catalysed by the same glycosyltransferases of the Golgi apparatus (Li and Ladisch, 1997): (1), GD3 synthase (*α*-2,8-sialyltransferase): (2), GM2/GD2 synthase (*β*-1,4-*N*-acetylgalactosaminyltransferase); (3), GD1b/GM1a synthase (*β*-1,3-galactosyltransferase); (4), GT1b/GD1a synthase (*α*-2,3-sialyltransferase); (5), GQ1b/GT1a synthase (*α*-2,8-sialyltransferase). For each enzyme, accession numbers according to the NCBI protein bank are included.

). Each ganglioside is structurally more complex than its precursor molecule, and the stepwise addition of monosaccharide or sialic acid residues by specific membrane-bound glycosyltransferases in the Golgi apparatus is catalysed by the same glycosyltransferases in both pathways (van Echten and Sandhoff, 1993).

Our previous studies have characterised ganglioside expression in human NB, a paediatric malignancy of neural crest origin (Wu *et al*, 1986; Valentino *et al*, 1990; Kaucic *et al*, 2001; Hettmer *et al*, 2003). We have linked alterations in the expression of complex ‘b’ pathway gangliosides (CbG) downstream of GD1b/GM1a synthase (GD1b, GT1b and GQ1b) to differences in the biological phenotype and clinical behaviour that can be used to predict patient outcome in NB (Hettmer *et al*, 2003). Further, recent experimental evidence suggests that GD1b, GT1b and GQ1b can modulate a number of biological processes that are believed to counteract malignant transformation and progression, including tumour cell proliferation, host immune function and signal transduction mechanisms (Hynds *et al*, 1995; Fukumoto *et al*, 2000; Kanda *et al*, 2001; Kanda and Watanabe, 2001). We hypothesise that high CbG content may contribute to reduced tumour aggressiveness, and that alteration of CbG expression might be a potential therapeutic target in NB.

One pharmacological agent that is well known to alter cellular ganglioside metabolism and successfully used in the oral maintenance therapy of disseminated NB is retinoic acid (Matthay *et al*, 1999). Previous *in vitro* studies have shown that morphological signs of neuronal differentiation in response to retinoic acid are accompanied by an increase in total ganglioside content and a relative increase in the expression of certain complex gangliosides of both the ‘a’ and ‘b’ pathway in NB cells (Li and Ladisch, 1992; Rebhan *et al*, 1994), as well as in normal and transformed embryonal cells (Levine and Flynn, 1986; Rizzo *et al*, 1995; Liour *et al*, 2000). On the basis of these findings, we have undertaken a comprehensive analysis of retinoic acid-induced changes in ganglioside expression and metabolism in human NB cell lines.

Here, we demonstrate that low CbG levels in some NB cell lines are analogous to the ganglioside pattern observed in clinically and biologically unfavourable NB tumours, providing an *in vitro* model to study pharmacological agents that might alter the metabolism of complex ganglioside subspecies. We further demonstrate that treatment with retinoic acid markedly enhances the activity of GD1b/GM1a synthase, resulting in increased expression of the complex gangliosides downstream of this enzyme, namely GD1b and GT1b (CbG), and GM1a and GD1a (CaG). Our results thus identify a specific effect of retinoic acid on the expression and biosynthesis of complex gangliosides in NB cells.

## MATERIALS AND METHODS

### Cell culture

Human NB cell lines LAN-1, LAN-5, SMS-KCN and SMS-KCNR were derived from three children with disseminated NB and have been previously described. SMS-KCN and SMS-KCNR were established from the same patient, from the primary tumour and bone marrow at relapse, respectively (Kohl *et al*, 1983; Reynolds *et al*, 1986; Rettig *et al*, 1987).

LAN-1 and LAN-5 were maintained in Waymouth's MB 752/1 medium (GIBCO, Grand Island, NY, USA), and SMS-KCN and SMS-KCNR in RPMI 1640 medium (GIBCO, Grand Island, NY, USA). The medium was supplemented with 2 mM L-glutamine and 10% heat-inactivated foetal calf serum (GIBCO, Grand Island, NY, USA), respectively. Cells were grown in adherent monolayer cultures using 75-cm^2^ and 175-cm^2^ cell culture flasks (Becton Dickinson, Franklin Lakes, NJ, USA) at 37°C in a 5% CO_2_ atmosphere in a humidified incubator.

Retinoic acid (all-*trans* or 13-*cis*; Sigma Chemical Co., St Louis, MO, USA) was dissolved in ethanol to a concentration of 10^−2^ M and kept as stock solution for up to 4 weeks at −70°C, protected from light. For each experiment, stock solutions were diluted with the growth medium to a final concentration of 5 or 10 *μ*M retinoic acid. These concentrations are equivalent to the serum levels effective in the oral maintenance therapy of disseminated NB with 13-*cis* retinoic acid (13-*cis* RA) (7.2±5.3 *μ*M) (Reynolds and Lemons, 2001). The final concentration of ethanol in the culture medium was ⩽0.2% (v v^−1^).

Cells were seeded at 7 × 10^4^ cells cm^−2^. After 24 h, the medium was replaced with a medium containing retinoic acid. Culture medium was replaced with fresh medium containing retinoic acid every other day. Untreated control cells were grown with and without ethanol. Cells were harvested after 5–7 days of exposure to retinoic acid, followed by ganglioside purification or extraction of crude membrane extracts, as described below. Viable cells were counted using trypan blue dye exclusion and the cellular protein content was estimated by the Bio-Rad D_C_ Protein Assay (Bio-Rad Laboratories, Hercules, CA, USA), using a bovine serum albumin standard.

### Ganglioside purification and quantification

Methods for extraction and purification of gangliosides were previously described (Ladisch and Gillard, 1985). Briefly, total lipid extracts of cell pellets were obtained by extracting the lyophilised cell pellets twice with chloroform/methanol (1 : 1 v v^−1^) at 4°C with stirring. Gangliosides were isolated by partitioning the dried total lipid extract in diisopropyl ether/1-butanol/17 mM aquaeous NaCl (6 : 4 : 5 v v^−1^). Gangliosides, in the lower aqueous phase, were further purified by Sephadex G-50 gel filtration.

High-performance thin layer chromatography (HPTLC) analysis of gangliosides (Ledeen and Yu, 1982) was performed using 10 × 10- or 10 × 20 cm precoated silica gel HPTLC plates (Merck, Darmstadt, Germany). The plates were developed in chloroform/methanol/0.25% CaCl_2_·H_2_O (60 : 40 : 9 by volume), and the gangliosides were stained with resorcinol. Absolute cellular ganglioside content was determined by HPTLC densitometry using known concentrations of human brain gangliosides (HBG) as the standard. Radioactive products were visualised by exposure of HPTLC plates to an X-ray film (BioMax, Kodak).

Individual gangliosides were identified using a human brain ganglioside standard or ^14^C-labelled rat brain gangliosides. The area under the peak generated by each ganglioside band was integrated after the plates/films were scanned (Microtek ScanMaker 5), and the relative percentage was calculated. To categorise individual ganglioside species, we used a previously established classification scheme based on biosynthetic pathways (Hettmer *et al*, 2003). Structurally complex molecules downstream of GD1b/GM1a synthase (*β*-1,3-galactosyltransferase) were designated complex ‘a’ gangliosides (CaG: GM1a, GD1a and GT1a) and complex ‘b’ gangliosides (CbG: GD1b, GT1b and GQ1b), respectively.

### Glycosyltransferase assays

The enzymatic activities of *α*-2,8-sialyltransferase (GD3 synthase), *β*-1,4-*N*-acetylgalactosaminyltransferase (GD2 synthase) and *β*-1,3-glactosyltransferase (GD1b synthase) were measured in membrane extracts from retinoic acid-treated and control cells by incorporation of radiolabelled sugars into ganglioside precursors (Kemp and Stoolmiller, 1976; Pohlentz *et al*, 2000).

#### Preparation of crude membrane extracts

Crude membrane fractions, used as the source of enzymes, were prepared by suspending cell pellets in five volumes of cold 0.25 M sucrose containing 0.01 M 2-mercaptoethanol. Cells were disrupted using eight cycles of freezing in liquid nitrogen and thawing in a water bath at 37°C. After centrifugation at 2000 **g** for 10 min to remove cellular debris, the supernatant fractions were centrifuged at 10 000 **g** for 10 min and then sedimented at 100 000 **g** for 1 h (Kemp and Stoolmiller, 1976). The sedimentable fractions were resuspended in unbuffered sucrose (0.25 M) and the amount of membrane protein was determined using the Bio-Rad D_C_ Protein Assay (Bio-Rad Laboratories, Hercules, CA, USA).

#### Processing of glycosyltransferse assays

Triton X-100 (50 *μ*g, Sigma Chemical Co., St Louis, MO, USA), ganglioside precursors and radiolabelled sugar donors in chloroform/methanol (1 : 1 v v^−1^) were transferred to 1 ml conical glass tubes and the solvent was evaporated under a stream of nitrogen. The corresponding buffer mixture and unlabelled sugar donor were added, the mixture was shaken vigorously, and each sample was sonicated for 30 s. Reaction mixtures were completed by addition of 50–150 *μ*g membrane protein to a final reactive volume of 50 *μ*l and shaken gently. After incubation at 37°C for 1 h, reactions were terminated by addition of 1 ml chloroform/methanol (2 : 1 v v^−1^) (Kemp and Stoolmiller, 1976; Pohlentz *et al*, 2000).

*α-2,8-sialyltransferase (GD3 synthase)* activity was determined using 30 nmol GM3 (Sigma Chemical Co., St Louis, MO, USA), 0.01 *μ*Ci ^14^C-labelled CMP sialic acid ([sialic-4,5,6,7,8,9-^14^C], 150 mCi mmol^−1^), 10 nmol ‘cold’ CMP sialic acid, 2.5 *μ*mol of MES buffer (pH 5.9), 2.5 *μ*mol of KCl, 0.1 *μ*mol of MnCl_2_ and 0.3 *μ*mol of MgCl_2_.

To assess *β-1,4-N-acetylgalactosaminyltransferase (GD2 synthase)* activity, 30 nmol GD3 (Matreya Inc., Pleasant Gap, PA, USA), 0.01 *μ*Ci ^3^H]-labelled UDP *N*-acetyl-D-galactosamine ([galactosamine-1-^3^H(*N*)], 5 Ci mmol^−1^), 14 nmol ‘cold’ *N*-acetyl-D-galactosamine, 2.5 *μ*mol MES (pH 6.5) and 1.5 *μ*mol MnCl_2_ were used.

For evaluation of *β-1,3-glactosyltransferase (GD1b synthase)* activity, the reaction mixture contained 10 nmol GD2 (Sigma Chemical Co., St Louis, MO, USA), 0.01 *μ*Ci ^14^C-labelled UDP galactose ([galactose–^14^C](U), 250 mCi mmol^−1^), 20 nmol ‘cold’ galactose, 2.5 *μ*mol MES (pH 6.5) and 2.1 *μ*mol MnCl_2_.

For each glycosyltransferase assay, buffer mixtures containing MES (Calbiochem, San Diego, CA, USA), KCl, MnCl_2_ and MgCl_2_ (Sigma Chemical Co., St Louis, MO, USA) were freshly prepared. Radiolabelled and ‘cold’ sugar donors were obtained from Perkin–Elmer Life Sciences Inc. (Boston, MA, USA) and Calbiochem (San Diego, CA, USA), respectively. All enzyme assays were performed in duplicates with the appropriate blank.

#### Separation of lipid-soluble reaction products

Lipid-soluble compounds in the reaction mixture were isolated by passing them through columns of Sephadex G25 Superfine (1 × 3–5 cm in 150 mm Pasteur pipettes, stuffed with glass wool). Sephadex G25 Superfine (Sigma Chemical Co., St Louis, MO, USA) was suspended in water (5 g per 60 ml), stored overnight at 4°C and shaken vigorously. Before pouring the columns, the gel was brought to room temperature and washed twice with the original volume of methanol (⩾30 min each time). One-third of the supernatant was decanted, the mixture was shaken vigorously to obtain a homogenous solution and the Pasteur pipettes were filled to the top. After the solvent had drained, the gel was overlayed with 1.5 ml methanol and equilibrated with chloroform/methanol/water (120 : 60 : 9 v v^−1^). The stopped samples were applied to the columns, the sample tubes washed with 1 ml chloroform/methanol/water (120 : 60 : 9 v v^−1^) and elution of the products was completed by adding 2 ml of the latter (Pohlentz *et al*, 2000).

#### Determination of glycosyltransferase activities and product identification

To measure the rate of incorporation of radiolabelled sugars, the column effluents were transferred in part (40%) to glass scintillation vials, evaporated to dryness under a stream of nitrogen, and dissolved in 10 ml Betafluor (National Diagnostics, Mannville, NJ, USA). Radioactivity (measured as d.p.m.) was determined in a Beckman LS6500 liquid scintillation counter. Quench correction was accomplished using the AutoDPM program. Enzyme activity (pmol h^−1^ mg protein^−1^) was calculated from the ratio of the products and the radioactivity of the total sample. The remainder of the sample was used to confirm the identity and the purity of the product by TLC autoradiography.

### Statistical analysis

The relative expression of individual gangliosides and ganglioside subsets (CbG and CaG) in each of the four cell lines studied were expressed as the mean±s.e.m. for three to five separate experiments. For the three cell lines that were responsive to retinoic acid, the relative ganglioside content was similarly reported as the mean±s.e.m. and, alternatively, the per cent of the expression of parallel control cells for two separate experiments per cell line. Data from six separate experiments involving three different cell lines were normalised by relating results in each experimental group to their respective controls by expressing the experimental (exp) levels as a per cent of the control (ctrl) levels (exp/ctrl × 100). Control values of zero were set to 1 before the computation. To test the hypothesis that experimental values differed from controls, we employed a *t*-test to examine whether the experimental values differed from 100%. The same method was used to compare glycosyltransferase activities among retinoic acid-treated and control cells in retinoic acid-responsive cell lines (LAN-1, LAN-5, SMS-KCNR). Results were considered statistically significant if the *P*-value was less than 0.050.

To examine whether experimental values differed from controls in studies of 13-*cis* RA, mean treatment values were compared to control values (no treatment with 13-*cis* RA) using a *t*-test.

## RESULTS

### Ganglioside expression in human NB cell lines

The impetus for this study were our findings (Hettmer *et al*, 2003) and those of others (Schengrund *et al*, 1985; Sung *et al*, 1995; Yates *et al*, 1999), suggesting that differences in tumour tissue CbG (GD1b, GT1b and GQ1b) expression are linked to the clinical and biological behaviour of NB and other tumours. In order to obtain an *in vitro* model that permits a more dynamic view of NB ganglioside biosynthesis, we analysed ganglioside expression in four well-described NB cell lines, LAN-1, LAN-5, SMS-KCNR and SMS-KCN.

**Figure 2:**
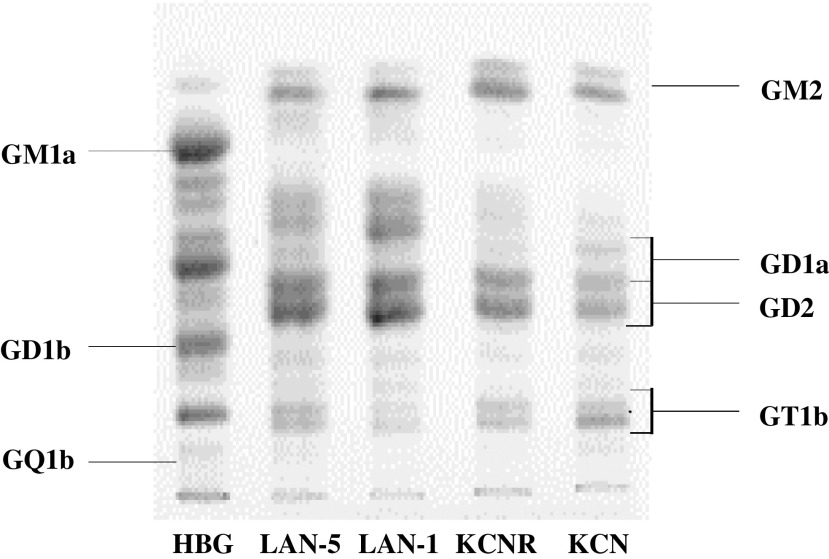
Ganglioside expression in four human NB cell lines (LAN-5, LAN-1, SMS-KCNR, SMS-KCN). Human brain gangliosides (HBG) were employed as a standard. GD2, GD1a and GT1b are doublets in tumour samples. The lower GD1a band and the upper GD2 band overlap.

To analyse ganglioside expression, we grew NB cells to 80–90% confluence, then extracted total cellular gangliosides and separated them by HPTLC (Figure 2

). Gangliosides were measured (i) as absolute cellular ganglioside content (nmol lipid-bound sialic acid (LBSA) 10^8^ cells^−1^) and (ii) as relative percentages of individual gangliosides out of total cellular gangliosides. Absolute cellular ganglioside content differed widely among the NB cell lines studied (69 nmol LBSA 10^8^ cells^−1^ for SMS-KCN, 57 nmol LBSA 10^8^ cells^−1^ for LAN-5, 30 nmol LBSA 10^8^ cells^−1^ for LAN-1 and 15 nmol LBSA 10^8^ cells^−1^ for SMS-KCNR). Nevertheless, there were clear similarities in the relative ganglioside content of the four cell lines (Table 1

**Table 1:** Ganglioside expression in neuroblastoma cell lines

	**Ganglioside expression (% of total cellular gangliosides)^a^**			
	**LAN-5**	**LAN-1**	**SMS-KCNR**	**SMS-KCN**
GM3	0±0	2±1	0±0	1±0
				
*‘a’ pathway*				
GM2	35±8	35±12	22±4	34±5
GM1a	2±1	1±0	1±0	2±1
GD1a	2±0	0±0	3±0	17±5
CaG	4±1	1±0	4±0	18±5
				
*‘b’ pathway*				
GD3	8±3	22±4	7±2	1±0
GD2	44±5	36±8	60±6	26±4
GD1b	2±1	2±1	2±1	2±1
GT1b	8±2	2±1	7±3	19±3
GQ1b	0±0	0±0	0±0	0±0
CbG	10±3	4±2	8±3	21±4

a Values represent means±s.e.m. of three independent experiments, except for LAN-5 which consisted of five independent experiments.

). The most prominent components were the ‘b’ pathway disialoganglioside GD2 (26–60%) and the ‘a’ pathway monosialoganglioside GM2 (22–35%). Complex ‘b’ pathway gangliosides represented only minor components (⩽10%) in three of the four NB cell lines studied (10% in LAN-5, 4% in LAN-1 and 8% in SMS-KCNR), and were composed of the disialylated molecule GD1b (2%) and the trisialylated ganglioside GT1b (2–8%). The fourth cell line, SMS-KCN, contained 21% CbG (2% GD1b and 19% GT1b). There were no detectable amounts of the most complex molecule, GQ1b, in any of the cell lines studied.

The prominent position of GD2 as a major ganglioside component in the NB cell lines studied (44% in LAN-5, 36% in LAN-1, 60% in SMS-KCNR and 26% in SMS-KCN) mirrors the ubiquitously high expression of GD2 that characterizes NB tumours (Wu *et al*, 1986). Importantly, the low CbG expression observed in each of the cell lines is also analogous to the CbG levels found in clinically and biologically unfavourable NB tumours (Hettmer *et al*, 2003). We therefore used these four NB cell lines as an *in vitro* model to determine whether retinoic acid, as a pharmacological agent, might alter the expression and biosynthesis of CbG.

### Retinoic-acid induced changes in CbG expression in NB cell lines

Retinoids are a class of vitamin A derivatives that differ in their isomeric conformations, and include all-*trans* retinoic acid (ATRA), 9-*cis* retinoic acid and 13-*cis* RA. Owing to its superior pharmacokinetics (higher and more sustained plasma levels), 13-*cis* RA is the isomer that is primarily used clinically. However, 13-*cis* RA isomerises *in vivo* to both ATRA and 9-*cis* retinoic acid, which are believed to be the active compounds *in vivo* in nonocular tissue (Lovat *et al*, 1994). Since retinoic acid treatment has been associated with changes in the ganglioside complement of various cell types (Li and Ladisch, 1992; Rebhan *et al*, 1994; Liour *et al*, 2000), and the oral maintenance therapy of disseminated NB with 13-*cis* RA results in improved event-free survival (Matthay *et al*, 1999), we sought to specifically explore the effect of retinoic acid on CbG expression. We, therefore, first treated NB cells *in vitro* with 10 *μ*M ATRA, then evaluated ATRA-induced changes in the composition of individual gangliosides and in total cellular ganglioside content in LAN-1, LAN-5, SMS-KCNR and SMS-KCN cells (Table 2

**Table 2:** Effect of ATRA on neuroblastoma cell ganglioside expression

	**Relative ganglioside expression (% of total cellular gangliosides)^a^**						**Absolute ganglioside expression (nmol 10^−8^ cells^−1^)**		
	**GD1b+GT1b (CbG)**	**GD2**	**GD3**	**GD1a+GM1a (CaG)**	**GM2**	**GM3**	**Total**	**CbG^b^**	**CaG^b^**
*LAN-1*									
Ctrl	3±2	29±1	18±1	1±0	48±2	3±1	30	0.9	0.3
ATRA	13±3	29±1	6±5	8±2	44±5	1±0	70	9.1	5.6
									
*LAN-5*									
Ctrl	6±4	48±3	5±2	4±2	38±6	0±0	57	3.4	2.3
ATRA	22±5	32±3	3±1	9±3	35±4	0±0	112	24.6	10.1
									
*SMS-KCNR*									
Ctrl	10±5	64±9	8±4	4±1	16±1	0±0	15	1.5	0.6
ATRA	40±3	27±2	1±1	30±1	5±4	0±0	94	37.6	28.2
									
*SMS-KCN*									
Ctrl	23±8	21±2	1±0	24±11	31±7	1±0	69	15.9	16.6
ATRA	23±4	11±1	6±5	18±6	43±6	6±4	50	11.5	9.0

a Values represent means±standard errors of two independent experiments.

b Calculated from the absolute total ganglioside content and the mean relative content of CaG or CbG.

ATRA=all-*trans* retinoic acid; Ctrl=control; CbG=complex ‘b’ pathway gangliosides; CaG=complex ‘a’ pathway gangliosides.

). Strikingly, ATRA induced a shift from synthesis of simpler gangliosides towards more complex species within both the ‘a’ and ‘b’ pathways in three of the four cell lines (LAN-1, LAN-5 and SMS-KCNR). In these three cell lines, the redistribution towards complex gangliosides included increased relative expression of GD1b and GT1b (‘b’ pathway) and GM1a and GD1a (‘a’ pathway), compounded by a substantial increase in total cellular ganglioside content, making the increase in the absolute content of CbG and CaG even more striking (Table 2). In LAN-5 cells for example, the ATRA-induced changes in cellular ganglioside expression included a 3.7-fold increase in CbG content (from 6 to 22%), a 2.2-fold increase in CaG content (from 4 to 9%) and a two-fold increase in absolute ganglioside content (from 57 to 112 nmol 10^8^ cells^−1^). SMS-KCN, the cell line with the highest baseline level of CbG, was the only cell line of the four which was not responsive to ATRA treatment; specifically, the change in complex ganglioside content was minimal (23 *vs* 23% CbG and 24 *vs* 18% CaG in controls compared to ATRA-treated cells), as was the change in total cellular ganglioside expression (69 *vs* 50 nmol 10^8^ cells^−1^). When normalised data from the three responsive cell lines were analysed together (Table 3

**Table 3:** Relative changes in neuroblastoma cell ganglioside expression in ATRA-responsive cell lines

		**Ganglioside expression in ATRA-treated cells (% of control)**					
	**Exp^a^**	**GD1b+GT1b (CbG)**	**GD2**	**GD3**	**GD1a+GM1a (CaG)**	**GM2**	**GM3**
LAN-1	a	250	96	6	1000	104	33
	b	1500	104	53	600	80	50
							
LAN-5	a	300	57	67	220	97	100
	b	850	76	57	300	91	100
							
SMS-KCNR	a	740	38	25	967	50	100
	b	307	45	0	750	7	100
							
Mean±s.e.m.^b^		658±202	69±11	35±12	640±137	72±15	80±13
*P*-value^c^		0.037	**0.040**	**0.002**	**0.010**	0.117	0.180

a For each cell line, two separate experiments (a and b) were analysed.

b Mean±s.e.m. of normalised values for all three cell lines (two experiments per cell line; six experiments total). Bold values are statistically significant (*P*<0.050).

c *t*-tests.

ATRA=all-*trans* retinoic acid; CbG=complex ‘b’ pathway gangliosides; CaG=complex ‘a’ pathway gangliosides.

), ATRA treatment clearly resulted in significant increases in the cellular content of CbG (658±202% of control, *P*=0.037) and CaG (640±137% of control, *P*=0.010), and, concomitantly, significant decreases in the cellular content of GD2 (69±11% of control; *P*=0.040) and GD3 (35±12% of control; *P*=0.002).

**Figure 3:**
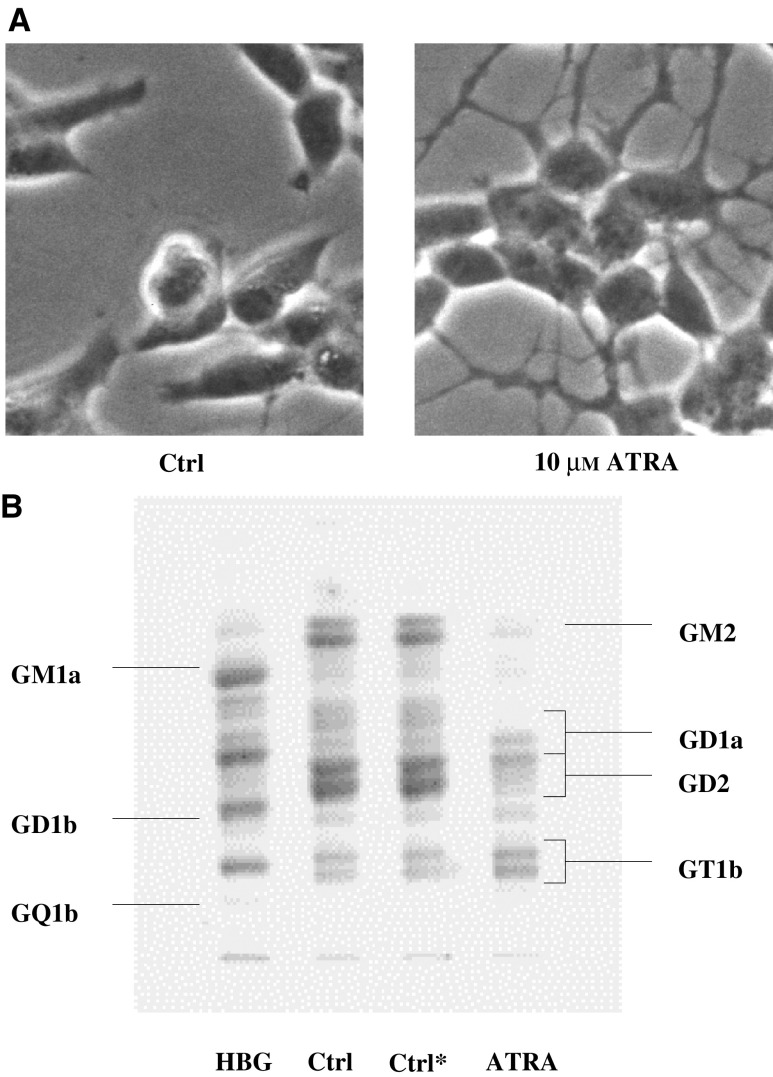
Effect of all-*trans* retinoic acid (ATRA) on cell morphology (**A**) and cellular ganglioside expression (**B**) in the human NB cell line SMS-KCNR. Cells were plated at 7 × 10^4^ cells and treated with ATRA 10 *μ*M on days 1–7. Photomicrograph (× 100) was obtained on day 6. Cells were harvested on day 7, and gangliosides were purified and separated by HPTLC (Ctrl, medium alone; Ctrl^*^, medium containing 0.1% ethanol; ATRA, medium containing 10 *μ*M ATRA). GD2, GD1a and GT1b are doublets in tumour samples. The lower GD1a band and the upper GD2 band overlap.

To illustrate these changes, Figure 3

summarises the ATRA-induced changes in cell morphology and ganglioside expression of the NB cell line SMS-KCNR. The morphological changes in response to ATRA, including rounding up of the cell body, extension of long processes with the appearance of neurites and cell aggregation into tight clusters (Figure 3A), are consistent with previous studies evaluating the differentiating effects of ATRA on NB cells (Li and Ladisch, 1992; Melino *et al*, 1997). Qualitative changes in the composition of cell-surface gangliosides accompanied morphological signs of differentiation in response to ATRA. The most prominent ganglioside components in untreated SMS-KCNR cells were the ‘b’ pathway ganglioside GD2 and the ‘a’ pathway ganglioside GM2. Treatment with ATRA increased the proportions of the CbG (GD1b and GT1b), as well as the CaG (GD1a and GM1a), such that these two subgroups predominated relative to other ganglioside species (Figure 3B). In view of the increase in total cellular ganglioside content (Table 2), this relative change resulted in significant absolute changes in the membrane expression of complex ganglioside molecules. Thus, the mean four-fold increase in relative CbG expression translated into a 25-fold increase in absolute cellular CbG expression in SMS-KCNR cells, indicating a striking accumulation of these biologically active molecules.

**Figure 4:**
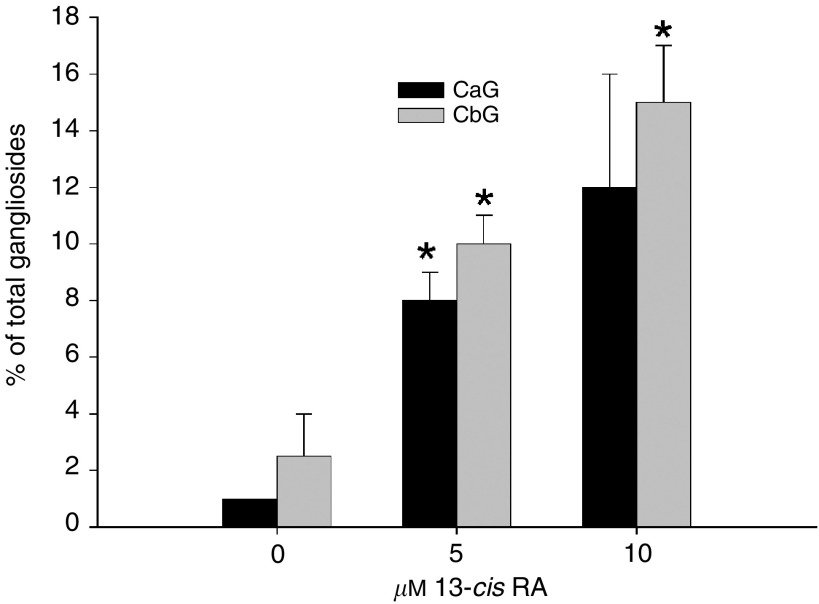
Effect of 13-*cis* retinoic acid (13-cis RA) on CbG (GD1b, GT1b; black bars) and CaG (GM1a, GD1a; grey bars) expression in LAN-1 human NB cells. Bars represent means±s.e.m. Ganglioside subsets are expressed as a percentage of total cellular gangliosides. Experimental and controls values were compared using a *t*-test (^*^P<0.050).

Since 13-*cis* RA is currently the only retinoid compound in clinical use to treat NB (Reynolds and Lemons, 2001), we also exposed one NB cell line, LAN-1, to 13-*cis* RA at two different concentrations (5 *μ*M, 10 *μ*M). We observed that 13-*cis* RA caused a concentration-related increase in the expression of structurally complex gangliosides in LAN-1 cells (Figure 4

). As with ATRA-treated cells, the increase in CbG expression was paralleled by a concurrent increase in CaG, suggesting a specific effect of retinoids on the activity of GD1b/GM1a synthase (*β*-1,3-galactosyltransferase), which initiates the synthesis of both CbG and CaG, from GD2 and GM2, respectively.

### Effect of retinoic acid on the activity of ganglioside glycosyltransferases in human NB cells *in vitro*

To delineate the metabolic basis for the ATRA-induced shift towards expression of complex gangliosides, we determined the effect of ATRA treatment on the activity of three key enzymes in the synthesis of these molecules. Precursor molecules enter ‘b’ pathway ganglioside biosynthesis through conversion of GM3 into GD3 by GD3 synthase (*α*-2,8-sialyltransferase). GD2/GM2 synthase (*β*-1,4-*N*-acetylgalactosaminyltransferase) and GD1b/GM1a synthase (*β*-1,3-glactosyltransferase) catalyse the two subsequent enzymatic reactions resulting in the synthesis of the most complex gangliosides in each pathway (van Echten and Sandhoff, 1993) (Figure 1). We measured the activities of GD3 synthase, GD2 synthase and GD1b synthase in crude membrane extracts from LAN-5, SMS-KCNR, SMS-KCN, using excess GM3, GD3 and GD2 as exogenously added ganglioside precursors.

When the retinoic acid-responsive LAN-5 or SMS-KCNR cells were exposed to 10 *μ*M ATRA for 6–7 days (Table 4

**Table 4:** Effect of ATRA on neuroblastoma (NB) cell glycosyltransferase activities

	**Enzyme activity (pmol h**^−1^**× mg protein)^a^**		
	**GD1b synthase**	**GD2 synthase**	**GD3 synthase**
*LAN-5*			
Ctrl	215	1416	572
ATRA	572	1384	669
			
*SMS-KCNR*			
Ctrl	172	518	364
ATRA	497	927	511
			
*SMS-KCN*			
Ctrl	186	1143	333
ATRA	283	769	405

a Activities of GD1b synthase, GD2 synthase and GD3 synthase in NB cells treated with 10 *μ*M all-*trans* retinoic acid (ATRA) are compared to untreated cells (Ctrl). Enzyme activities were determined by incorporation of radiolabelled sugars into ganglioside precursors. Values represent means from duplicate samples of one to two experiments.

ATRA=all-*trans* retinoic acid; Ctrl=control; CbG=complex ‘b’ pathway gangliosides; CaG=complex ‘a’ pathway gangliosides.

), we observed a marked increase (2.7–2.9-fold, *P*<0.050) in GD1b synthase activity. This was accompanied by a lesser (0–1.8-fold, *P*=0.198) increase in GD2 synthase activity, and only small changes in GD3 synthase activity (1.2–1.4, *P*=0.161). Thus, in both LAN-5 and SMS-KCNR cells, increased expression of complex gangliosides downstream of GD1b sythase was associated with a significant (over two-fold) increase in GD1b synthase activity. In contrast, in SMS-KCN cells, the cell line that was unresponsive to ATRA treatment, GD1b synthase activity was only slightly increased (1.5-fold) in ATRA-treated *vs* control cells.

Figure 5

**Figure 5:**
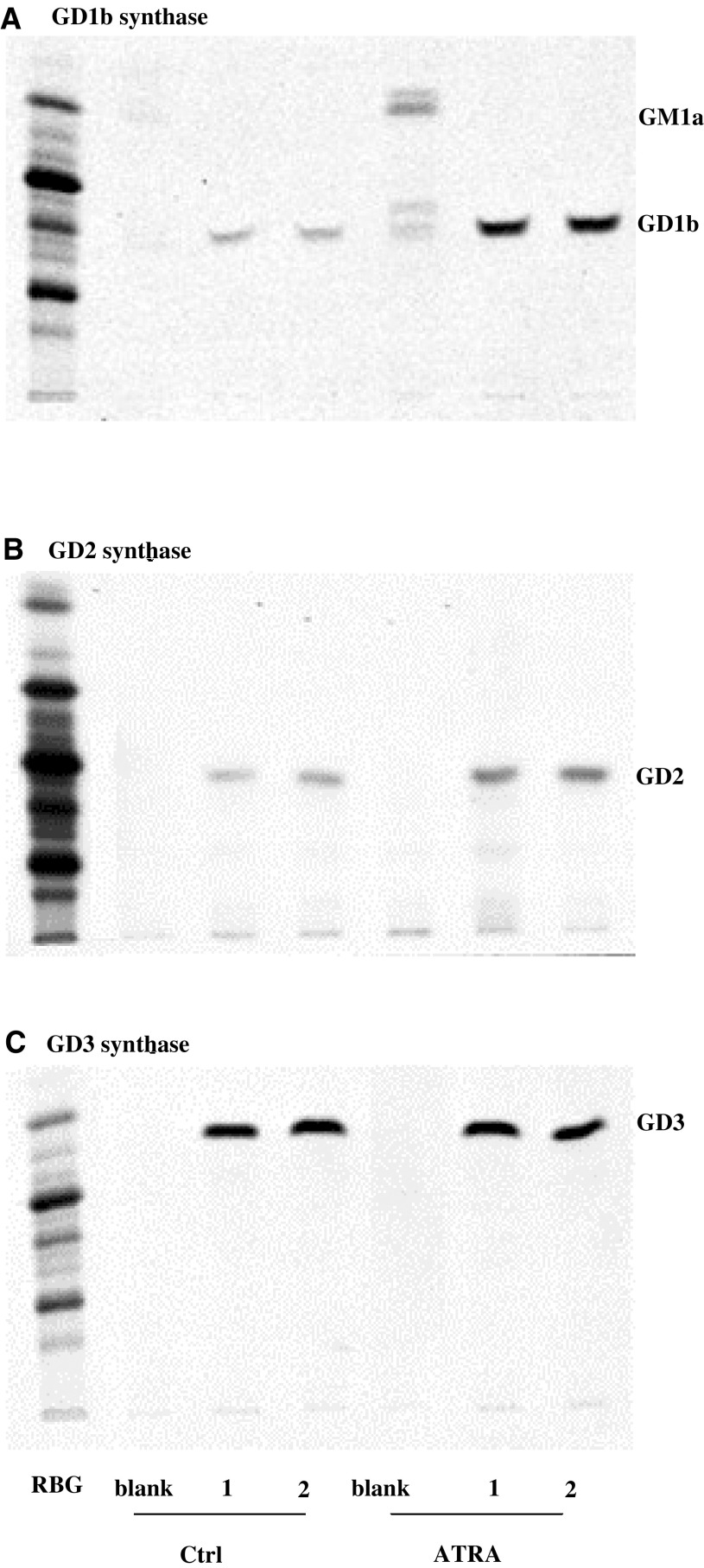
Effect of all-*trans* retinoic acid (ATRA) on the activities of GD1b synthase, GD2 synthase and GD3 synthase in the human NB cell line SMS-KCNR. HPTLC autoradiograms demonstrate reaction products from assays measuring activities of GD1b synthase (**A**), GD2 synthase (**B**) and GD3 synthase (**C**). Enzyme assays were performed in duplicate (1,2) with an appropriate ‘blank’ (Ctrl, medium alone; ATRA, 10 *μ*M).

illustrates the autoradiographic visualisation of enzyme products resulting from ATRA-induced changes in ganglioside metabolism in SMS-KCNR cells, confirming the major increase in GD1b synthase activity (Figure 5A), a comparatively minor increase in GD2 synthase activity (Figure 5B) and almost no change in GD3 synthase activity (Figure 5C). It is interesting to note that in the case of the ATRA-treated cell samples, the parallel blank (which does not contain exogenously added ganglioside precursors) also shows the synthesis of GD1b and GM1a from the trace amounts of endogenous gangliosides present in the cell pellet (Figure 5A), further confirming the substantial increase in the activity of GD1b/GM1a synthase following treatment with ATRA. Taken together, our data demonstrate that the increase in GD1b synthase activity is specifically associated with increased expression of complex ganglioside species, induced by ATRA in NB cells *in vitro*, and provides strong evidence that elevated CbG and CaG expression can be accounted for by a specific effect of ATRA on this enzyme.

## DISCUSSION

Treatment with retinoic acid is shown here to induce a dramatic shift from synthesis of simpler gangliosides towards predominant expression of structurally complex ‘a’ and ‘b’ pathway ganglioside molecules downstream of GD1b/GM1a synthase in some NB cell lines. The increase in total cellular ganglioside content combined with the relative changes in the proportion of complex gangliosides results in a significant increase in the absolute cellular content of these molecules. The findings are consistent with the previous observations of a dramatic increase in membrane expression and shedding of total cellular gangliosides and enhancement in GD1a and GT1b content in LAN-5 NB cells treated with retinoic acid (Li and Ladisch, 1992).

Among the four cell lines employed in this study, the NB cell line SMS-KCN appears to be biologically distinct. Three differences are evident. First, SMS-KCN cells consist of two morphologically distinct cell populations, one neuroblastic (N-type) and the second substrate-adherent (S-type) with epithelial or fibroblast-like morphology (Rettig *et al*, 1987), while LAN-1, LAN-5 and SMS-KCNR exhibit a uniform phenotype with small, round, neuroblastic cells that have short neuritic processes. Second, constitutive CbG content in SMS-KCN was higher (1.8- to 5.2-fold) than in the other cell lines studied. Third, treatment with retinoic acid did not induce the alterations in cellular ganglioside biosynthesis observed in the other cell lines. These differences in ganglioside metabolism do not have an obvious explanation, but it is possible that they are accounted for by constitutive differences between N-type and S-type cells.

One possible explanation for the retinoic acid-induced changes in ganglioside composition in LAN-1, LAN-5 and SMS-KCNR cells is an increased rate of synthesis of certain gangliosides. A previous study in embryonal carcinoma cells demonstrated enhanced activities of various ganglioside glycosyltransferases at different stages of retinoic acid-induced neuronal differentiation, and a parallel increase in mRNA expression, suggesting that retinoic acid activated the transcription of these glycosyltransferases or stabilised their expression (Osanai *et al*, 1997; Liour *et al*, 2000). Our data support that the retinoic acid-induced increase in the expression of complex gangliosides both within the ‘a’ and the ‘b’ pathway is attributable to an induction of GD1b/GM1a synthase (*β*-1,3-glactosyltransferase) activity. The retinoic acid-induced alterations in the ganglioside complement of NB cells seem to reflect, therefore, a specific effect on ganglioside anabolism, although, theoretically, alterations in ganglioside catabolism or shedding are also possible contributing factors. Increased expression of complex gangliosides is a well-described feature of normal embryonic neurogenesis and accelerated axonogenesis (Svennerholm *et al*, 1989; Hirschberg *et al*, 1996). Similar ganglioside changes to those we describe here have been reported in retinoic acid-treated Xenopus embryo cells, murine embryonal carcinoma cells and SHSY-5Y NB cells during neuronal maturation (Rebhan *et al*, 1994; Rizzo *et al*, 1995; Liour *et al*, 2000). Thus, predominant expression of complex gangliosides can be considered a biochemical marker of increasing neuronal differentiation. Further, the retinoic acid-induced changes in the ganglioside, complement of NB cell lines, are analogous to reported differences in CbG content among prognostically distinct categories of NB tumours (Schengrund *et al*, 1985; Schengrund and Shochat, 1988; Hettmer *et al*, 2003). Specifically, we previously found that among 74 human NB tumours, high (⩾35%) CbG expression (a) characterised nonprogressive *vs* progressive tumours (median 41 *vs* 18% of total gangliosides), and (b) was strongly predictive of a favourable outcome, even when other prognostic factors were considered (Hettmer *et al*, 2003). Related studies, associating low CbG levels in astrocytoma and medulloblastoma with higher histological grades and lower survival (Sung *et al*, 1995; Yates *et al*, 1999), further support the value of CbG in discriminating between different categories of malignant disease. In this context, the retinoic acid-induced increase in CbG expression in NB cells represents a transition into a ganglioside pattern which is associated with (i) neuronal differentiation in normal cells and (ii) clinically less-aggressive NB tumours, suggesting that CbGs may play a role in the biological behaviour of NB.

Tumour gangliosides of aberrant structure are generally believed to favour tumour progression (Hakomori, 1996), and a substantial body of previous experimental evidence associates the shedding of tumour cell surface gangliosides with increased tumorigenicity (Ladisch *et al*, 1987). The relationship between the concentration of shed GD2 in the plasma of NB-bearing patients and reduced long-term survival (Valentino *et al*, 1990) is consistent with this concept. There is also experimental evidence that suggests that the biological effects of tumour-derived gangliosides differ greatly depending on their carbohydrate and ceramide structures (Floutsis *et al*, 1989; Ladisch *et al*, 1994). Here we hypothesise that the significantly higher CbG levels in clinically favourable NB tumours (Hettmer *et al*, 2003) may lead to reduced NB tumour aggressiveness. Substantial experimental evidence corroborates the concept that the prognostic value of CbG reflects their functional involvement in the cellular processes that define the malignant behaviour of NB. Malignant transformation of murine epidermal cells in response to tumour-promoting phorbol esters is accompanied by a decrease in GT1b synthesis (Srinivas and Colburn, 1982). Proliferation of and IL-8 production by human metastatic melanoma cells is inhibited by GD1b, GT1b and GQ1b (Kanda *et al*, 2001). Platelet-derived growth factor (PDGF)-mediated cell growth and PDGF receptor activation in NB and glioma cells are inhibited by complex ganglioside species (Hynds *et al*, 1995). These gangliosides also modulate immunoglobulin synthesis in peripheral blood mononuclear cells (Kanda and Tamaki, 1998, 1999a and 1999b) and cause a shift from Th-2 to Th-1 cytokine production in PHA-stimulated T cells (Kanda *et al*, 2001). Finally, increased expression of GD1b and GT1b in rat pheochromocytoma cells transfected with GD3 synthase occurs in parallel with trk-A dimerisation (Fukumoto *et al*, 2000), which in turn is associated with improved prognosis in NB (Nakagawara *et al*, 1993). Taking these findings together, we hypothesise that CbG may impede tumour progression, either by acting directly on the tumour cell or by increasing the host resistance.

Similarly, increased tumour CbG expression might contribute to the success of retinoic acid in the maintenance therapy of disseminated NB. The beneficial role of retinoic acid is generally attributed to a reversal of the malignant state through differentiation of the tumour cells into more mature ganglion-like cells (Matthay *et al*, 1999; Reynolds and Lemons, 2001). Substantial experimental evidence suggests that complex gangliosides are biologically relevant molecules during cellular differentiation and development. First, outgrowth of cellular extensions, one aspect of neuronal differentiation, can be induced by treating NB cells *in vitro* with exogenous gangliosides, and the CbG species GT1b and GQ1b have been repeatedly described as the two most potent ganglioside molecules with differentiating properties (Tsuji *et al*, 1983; Leskawa and Hogan, 1985). Secondly, increased expression of GD1b and GT1b in murine NB cells transfected with GD3 synthase occurs in parallel with morphological signs of increased cellular differentiation (Kojima *et al*, 1994). Thirdly, absence of all gangliosides downstream of GM2/GD2 synthase (CbG+GD2 and CaG+GM2) in genetically altered mice with a disrupted gene for that enzyme is associated with decreased myelination and axon degeneration in the central and peripheral nervous system (Sheikh *et al*, 1999), although brain development and gross behaviour is normal (Takamiya *et al*, 1996). Finally, although retinoic acid-induced neurite outgrowth is unaffected by abrogation of cellular ganglioside synthesis in LAN-5 cells grown in a serum-supplemented environment (Li and Ladisch, 1997), reduction of cellular ganglioside synthesis is associated with decreased NGF-induced outgrowth of neuritic processes in a NB cell line grown in serum-free medium, and is reversible by exogenous addition of gangliosides (Rosner, 1998).

Previous findings and our demonstration of induction of CbG (and CaG) synthesis by retinoic acid underscore the potential role of complex gangliosides in cellular differentiation and other cellular functions important to reversion of the malignant phenotype. Consequently, the specific role of complex gangliosides compared to structurally simpler molecules in influencing cellular differentiation and other cellular functions in NB and other neuroectodermal tumours will be important to delineate.
